# SHAMAN: a user-friendly website for metataxonomic analysis from raw reads to statistical analysis

**DOI:** 10.1186/s12859-020-03666-4

**Published:** 2020-08-10

**Authors:** Stevenn Volant, Pierre Lechat, Perrine Woringer, Laurence Motreff, Pascal Campagne, Christophe Malabat, Sean Kennedy, Amine Ghozlane

**Affiliations:** 1 Hub de Bioinformatique et Biostatistique – Département Biologie Computationnelle, Institut Pasteur, USR 3756 CNRS, 28 Rue Du Docteur Roux, Paris, 75015 France; 2 Biomics – Département Génomes et Génétique, Institut Pasteur, 28 Rue du Docteur Roux, Paris, 75015 France

**Keywords:** Metagenomics, Differential analysis, Visualization, Web application

## Abstract

**Background:**

Comparing the composition of microbial communities among groups of interest (e.g., patients vs healthy individuals) is a central aspect in microbiome research. It typically involves sequencing, data processing, statistical analysis and graphical display. Such an analysis is normally obtained by using a set of different applications that require specific expertise for installation, data processing and in some cases, programming skills.

**Results:**

Here, we present SHAMAN, an interactive web application we developed in order to facilitate the use of (i) a bioinformatic workflow for metataxonomic analysis, (ii) a reliable statistical modelling and (iii) to provide the largest panel of interactive visualizations among the applications that are currently available. SHAMAN is specifically designed for non-expert users. A strong benefit is to use an integrated version of the different analytic steps underlying a proper metagenomic analysis. The application is freely accessible at http://shaman.pasteur.fr/, and may also work as a standalone application with a Docker container (aghozlane/shaman), conda and R. The source code is written in R and is available at https://github.com/aghozlane/shaman. Using two different datasets (a mock community sequencing and a published 16S rRNA metagenomic data), we illustrate the strengths of SHAMAN in quickly performing a complete metataxonomic analysis.

**Conclusions:**

With SHAMAN, we aim at providing the scientific community with a platform that simplifies reproducible quantitative analysis of metagenomic data.

## Background

Quantitative metagenomic techniques have been broadly deployed to identify associations between microbiome and environmental or individual factors (e.g., disease, geographical origin, etc.). Analyzing changes in the composition and/or in the abundance of microbial communities yielded promising biomarkers, notably associated with liver cirrhosis [1], diarrhea [2], colorectal cancer [3], or associated with various pathogenic [4] or probiotic effects [5] on the host.

In metataxonomic studies, a choice is made prior to sequencing in order to specifically amplify one or several regions of the rRNA (usually the 16S or the 18S rRNA genes for procaryotes/archaea and the ITS, the 23S or the 28S rRNA gene for eukaryotes) so that the composition of microbial communities may be characterized with affordable techniques.

A typical workflow includes successive steps: (i) OTU (Operational Taxonomic Unit) picking (dereplication, denoising, chimera filtering and clustering) [6], (ii) OTU quantification in each sample and (iii) OTU annotating with respect to a reference taxonomic database. This process may require substantial computational resources depending on both the number of samples involved and the sequencing depth. Several methods are currently available to complete these tasks, such as Mothur [7], Usearch [8], DADA2 [9] or Vsearch [10]. The popular application Qiime [11] simplifies these tasks (i to iii) and visualizations by providing a python-integrated environment. Schematically, once data processing is over, both a contingency table and a taxonomic table are obtained. They contain the abundance of OTUs in the different samples and the taxonomic annotations of OTUs, respectively. The data are normally represented in the standard BIOM format [12].

Statistical analysis is then performed to screen significant variation in microbial abundance. To this purpose, several R packages were developed, such as Metastats [13] or Metagenomeseq [14]. It is worth noticing that other approaches which were originally designed for RNA-seq, namely DESeq2 [15] and EdgeR [16], are also commonly used to carry out metataxonomic studies [17, 18]. They provide an R integrated environment for statistical modelling in order to test the effects of a particular factor on OTU abundance. Nevertheless using all of these different methods requires technical skills in Unix, R and experience in processing metagenomics data. To this end, we developed SHAMAN in order to simplify the analysis of metataxonomic data, especially for users who are not familiar with the technicalities of bioinformatic and statistical methods that are commonly applied in this field.

SHAMAN is an all-inclusive approach to estimate the composition and abundance of OTUs, based on raw sequencing data, and to perform statistical analysis of processed files. First, the user can submit raw data in FASTQ format and define the parameters of the bioinformatic workflow. The output returns, a BIOM file for each database used as a reference for annotation, a phylogenetic tree in Newick format as well as FASTA-formatted sequences of all OTUs that were identified. The second step consists in performing statistical analysis. The user has to provide a “target” file that associates each sample with one or several explanatory variables. These variables are automatically detected in the target file. An automatic filtering of the contingency matrix of OTUs may be activated in order to remove features with low frequency. Setting up the contrasts to be compared is also greatly simplified. It consists in filling in a form that orients the choices of users when defining the groups of interest. Several options to visualize data are available at three important steps of the process: quality control, bio-analysis and contrast comparison. At each step, a number of common visual displays are implemented in SHAMAN to explore data. In addition, SHAMAN also includes a variety of original displays that is not available in other applications such as an abundance tree to visualize count distribution according to the taxonomic tree and variables, or the logit plot to compare feature *p*-values in two contrasts. Figures may be tuned to emphasize particular statistical results (e.g., displaying significant features in a given contrast only, displaying intersections between contrasts), to be more specific (e.g. feature abundance in a given modality) or to improve the aesthetics of the graph (by changing visual parameters). Figures fit publication standards and the corresponding files can be easily downloaded.

Several web applications were developed to analyze data of metataxonomic studies, notably, FROGS [19], ASaiM [20], Qiita [21] as well as MetaDEGalaxy [22] for bioinformatic data processing, Shiny-phyloseq [23] for statistical analysis, Metaviz [24] and VAMPS2 [25] that make a particular focus on data visualization. While these interfaces propose related functionalities, the main specificity of SHAMAN is to combine of all these steps in a single user-friendly application. Last, SHAMAN may keep track of a complete analysis which may be of particular interest for matters of reproducibility.

## Material and method

**Fig. 1:**
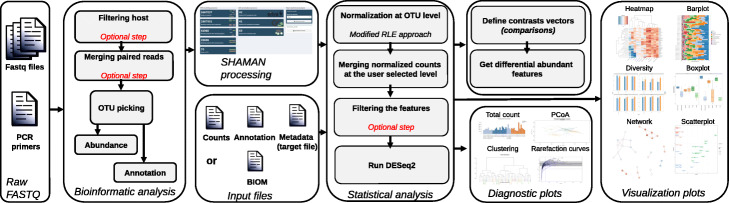
SHAMAN workflow. SHAMAN can start from raw reads or from processed data. In this last case, it needs at least three tables, the count matrix, the annotation table and the metadata which can either be provided into three different files or by using the BIOM format. The user can then select the variables of interest and add some batch effects. Then, contrast vectors can easily be defined and interactive visualizations are available

SHAMAN is implemented in R using the shiny-dashboard framework. The application is divided into three main components (Fig. 1): a bioinformatic workflow to process the raw FASTQ-formatted sequences, a statistical workflow to normalize and further analyse data, as well as a visualization platform. Users may run each component of the workflow independently or run the whole process from raw FASTQ data to visualization. SHAMAN provides, for each component, scores and figures that summarize the quality of data processing. When installed with Docker, SHAMAN has low computing resource requirements with a minimum 1Ghz processor, 1Gbyte of ram memory and 3.4Gbytes of disk.

### Bioinformatic workflow in SHAMAN

The metataxonomic pipeline implemented in SHAMAN relies on the Galaxy platform [26]. All calculations are remotely done on galaxy.pasteur.fr; this process is transparent to the user. The pipeline flows in the following manner:

1. Optional filtering of reads. It is worth noticing that previous studies, e.g. carried out on mosquito microbiota [27], showed that some non-annotated OTUs turned to be sequences of the host organism. To overcome such issues, the user can optionally filter out reads that align with the host genome and the PhiX174 genome (used as a control in Illumina sequencers). The latter task is performed with Bowtie2 v2.2.6 [28].
2. Quality of reads is checked with AlienTrimmer [29] v0.4.0, a software for trimming off contaminant sequences and clipping.
3. Paired-end reads are then merged with Pear [30] v0.9.10.1.
4. OTU picking, taxonomic annotation and OTU quantification are performed using Vsearch [10] v2.3.4.0, a software that was shown to be both accurate and efficient [6, 31]. The OTU picking process consists in five steps, i.e., dereplication, singleton removal, chimera detection, clustering and alignment. It follows the approach and default parameters previously described by the Uparse pipeline [8]. Input amplicons are aligned against the set of detected OTUs to create a contingency table that contains the number of amplicons assigned to each OTU. This step aims at refining OTU counts by including singletons that correspond to sequences with a reasonable amount of sequencing errors (i.e., <3%).
5. The taxonomic annotation of OTUs is performed based on various databases, i.e., with SILVA [32] rev. 132 SSU (for 16S, 18S rRNA genes) and LSU (for 23S and 28S rRNA genes), Greengenes [33] (for 16S, 18S rRNA genes) and Underhill rev. 1.6.1 [34], Unite rev. 8.0 [35] and Findley [36] for ITS rRNA sequences. These databases are kept up-to-date every two month with biomaj.pasteur.fr.
6. OTU annotations are filtered according to their identity with the reference [37]. Phylum annotations are kept when the identity between the OTU sequence and the reference sequence is ≥ 75%, ≥ 78.5% for classes, ≥ 82% for orders, ≥ 86.5% for families, ≥ 94.5% for genera and ≥ 98% for species. In addition, a taxonomic inference based on a naive Bayesian approach, RDP classifier [38] v2.12, is systematically provided. By default, RDP annotations are included whenever the annotation probability is ≥ 0.5. All the above-mentioned thresholds may be tuned by the user.
7. A phylogenetic analysis of OTUs is provided: multiple alignments are obtained with Mafft [39] v7.273.1, filtering of regions that are insufficiently conserved is made using BMGE [40] v1.12 and finally, FastTree [41] v2.1.9 is used to infer the phylogenetic tree. Based on the latter tree, a Unifrac distance [42] may be computed in SHAMAN to compare microbial communities.

The outcomes of the overall workflow are stored in several files: a BIOM file (per reference database), a phylogenetic tree as well as a summary file specifying the number of elements passing the different steps of the workflow. The data are associated to a key that is unique to a project. Such a key allows to automatically re-load all results previously obtained within a given project.

### Statistical workflow in SHAMAN

The statistical analysis in SHAMAN is based on DESeq2 which is a method to model OTU counts with a negative binomial distribution. It is known as one of the most accurate approach to detect differentially abundant bacteria in metagenomic data [17, 18]. Relying on robust estimates of variation in OTUs, the DESeq2 method has suitable performances even with datasets characterized by a relatively low number of observations per group (together with a high number of OTUs).

This method typically requires the following input files: a contingency table, a taxonomic table and a target file describing the experimental design. These data are processed to generate a meta-table that assign to each OTU, a taxonomic annotation and a raw count per sample.

#### Normalization

Normalization of the raw counts is one of the key issues when analyzing microbiome experiments. The uniformity of the sequencing depth is affected by sample preparation and dye effects [43]. Normalizing data is therefore expected to increase the accuracy of comparisons. It is done by adjusting the abundance of OTUs across samples. Four different normalization methods are currently implemented in SHAMAN. For the sake of consistency, all of these methods are applied at the OTU level.

A first method is the relative log expression (RLE) normalization and is implemented in the DESeq2 package. It consists in calculating a size factor for each sample, i.e., a multiplication factor that increases or decreases the OTU counts in samples. It is defined as the median ratio between a given count and the geometric mean of the corresponding OTU. Such a normalization was shown to be suited for metataxonomic studies [17]. In practice, many OTUs are found in a few samples only, which translate into sparse count matrices [14]. In this case, the RLE method may lead to a defective normalization - as only a few OTU are taken into account - or might be impossible if all OTUs have a null abundance in one sample at least. In the R package Phyloseq [44], the decision was made to calculate a modified geometric mean by taking the *n*-th root of the product of the non-zero counts, which is equivalent to replacing the null abundance by a count of 1. This approach might impact the normalization process when the input matrix is very sparse. As a consequence, we decided to include two new normalization methods : the *non-null* and the *weighted non-null* normalizations. They are modified versions of the original RLE so that they better account for matrix sparsity (number of elements with null values divided by the total number of elements). In the *non-null normalization* (1) cells with null values are excluded from the computation of the geometric mean. This method therefore takes all OTUs into account when estimating the size factor. In the second method that we coined the *weighted non-null normalization* (2), weights are introduced so that OTUs with a larger number of occurrences have a higher influence when calculating the geometric mean.

Assume that *C*=(*c*_*ij*_)_1≤*i*≤*k*;1≤*j*≤*n*_ is a contingency table where *k* and *n* are the number of features (e.g. OTUs) and the number of samples, respectively. Here, *c*_*ij*_ represents the abundance of the feature *i* in the sample *j*. The size factor of sample *j* is denoted by *s*_*j*_.

1 $$ s_{j}^{(1)} = median_{i} \frac{c_{ij} }{\left(\prod_{k\in S_{i}} c_{ik} \right)^{1/n_{i}}},   $$

2 $$  s_{j}^{(2)} = w.median_{i} \frac{c_{ij} }{\left(\prod_{k\in S_{i}} c_{ik} \right)^{1/n_{i}}},  $$

where *S*_*i*_ stands for the subset of samples with non null values for the feature *j* and *n*_*i*_ is the size of this subset. The function *w*.*median* corresponds to a weighted median.

An alternative normalization technique is the *total counts* [45] which is convenient for highly unbalanced OTU distribution across samples.

Using a simulation-based approach, we addressed the question of the performance of the *non-null* and the *weighted non-null normalization* techniques when the matrix sparsity and the number of observations increase. We compared these new methods to those normally performed with DESeq2 and Phyloseq. To do so, we normalized 500 simulated matrices using the function makeExampleDESeqDataSet of DESeq2 with varied sparsity levels (i.e., 0.28, 0.64 and 0.82) and different numbers of observations (i.e., *n*= 4, 10 and 30). We calculated the average coefficient of variation (CVmean) [46] for each normalization method (Fig. S1). Considering that these OTUs are assumed to have relatively constant abundance within the simulations, the coefficient of variation is expected to be lower when the normalization is more efficient. In this simulation-based comparison, the *non-null* and the *weighted non-null* normalization methods exhibited a lower coefficient of variation as compared to the other methods, when sparsity in the count matrix is high and the number of observations is increased. These differences were clear especially when comparing the normalization methods used in DESeq2 and Phyloseq to the *weighted non-null normalization* (sparsity ratio of 0.28, 0.64 and 0.82, with 30 samples; t-tests *p*<0.001) (Fig. S1).

#### Contingency table filtering

In metataxonomic studies, contingency tables are often very sparse and after statistical analysis, some differences associated with *p*-values <0.05 are not necessarily of great relevance, due to violated assumptions. This may arise when a feature, distributed in many samples with a low abundance, is slightly more abundant in one group of comparison. These artifacts are generally excluded by DESeq2 with an independent filtering. Furthermore, if a feature is found in high abundance in a few samples only (and count is 0 in the other samples), it may lead to non-reliable results. Such distributions may also affect the normalization process as well as the dispersion estimates. In order to avoid misinterpretation of results, we propose an optional extra-step of filtering, by excluding features characterized by a low abundance and/or a low number of occurrence in samples (e.g. features occurring in less than 20% of the samples). To set a by-default abundance threshold, SHAMAN searches for an inflection point at which the curve between the number of observations and the abundance of features changes from being linear to concave. This process is performed with linear regression in the following manner:

1. We define *I* the interval $\left [ min_{j} \left (\sum _{i} c_{ij}\right) ; \frac {\sum _{ij} c_{ij}}{k} \right ]$.
2. For each *x*∈*I*, we compute *h*(*x*) defined as the number of observations with a total abundance higher than *x*.
3. We compute the linear regression between *h*(*x*) and *x*.
4. The intercept is set as the default threshold.

(see Appendix 1 for more information). This extra-filtering is more stringent than the DESeq2 process and normally results in decreased computation time. The impact of filtering steps may be visually assessed with plots displaying features that will be included in the analysis and those that will be discarded.

#### Statistical modelling

The statistical model relies on the variables that are loaded in the file of experimental design. By default, all variables are included in the model but the end-user can edit this selection and further add interactions between variables of interest. In addition, other variables such as batches or clinical data (e.g., age, sex, etc.) may be used as covariates. SHAMAN then automatically checks whether the model is statistically suitable (i.e., whether the parameters may be estimated properly). When it is not the case, a warning message appears and a “how to” box proposes a practical way to solve the issue. In SHAMAN, statistical models may be fitted at any taxonomic levels: normalized counts are summed up within a given taxonomic level.

To extract features that exhibit significant differential abundance (between two groups), the user must define a contrast vector. Both a guided mode and an expert mode are available in SHAMAN. In the guided mode, the user specifies the groups to be compared using a dropdown menu. This mode is only available for DESeq2 v1.6.3 which is implemented in the DESeq2shaman package (https://github.com/aghozlane/DESeq2shaman). In advanced comparisons, the user may define a contrast vector by specifying coefficients (e.g., -1, 0, 1) assigned to each variable.

### Visualization in SHAMAN

After running a statistical analysis, many displays are available:

- Diagnostic plots (such as barplots, boxplots, PCA, PCoA, NMDS and hierarchical clustering) help the user examine both raw and normalized data. For instance, these plots may reveal clusters, sample mislabelling and/or batch effects. Scatterplots of size factors and dispersion estimates (i.e., estimates that are specific to DESeq2) are useful when assessing both the relevance and robustness of statistical models. PCA- and PCoA-plots associated with a PERMANOVA test may be used as preliminary results in the differential analysis as they may reveal global effects among groups of interest.
- All significant features are gathered in a table including, the base mean (mean of the normalized counts), the fold change (i.e., the factor by which the average abundance changes from one group to the other), as well as the corresponding adjusted *p*-values. The user may view tables for any contrasts and can export them into several formats. Volcano plots and bar charts of *p*-values and log2 fold change are also available in this section.
- A global visualization section provides a choice of 9 interactive plots such as barplots, heatmaps and boxplots to represent differences in abundance across groups of interest. Diversity plots display the distribution of various diversity indices: alpha, beta, gamma, Shannon, Simpson and inverse Simpson. Scatterplots and network plots may reveal associations between feature abundance and other variables from the target file. To explore variations of abundance across the taxonomic classification, we included an interactive abundance tree and a Krona plot [47]. Rarefaction curves are of great use to further consider the number of features in samples with respect to the sequencing depth.
- In the comparison section, plots of comparisons among contrasts may be created. Several options are available such as, Venn diagram or upsetR graph [48] (displaying subsets of common features across contrast), heatmap, a logit plot [49] (showing the log2 fold-change values in each feature), a density plot and a multiple Venn diagram to summarize the number of features captured by each contrast. All these graphs can be exported into four format (eps, png, pdf and svg).

### User case datasets

To illustrate how SHAMAN operates, we analysed two datasets. The first dataset originates from a mock sequencing we performed on purpose (a standard practice when assessing metagenomics methods). The second dataset is publicly available and originates from a published study (the afribiota dataset, [50]). The latter dataset was collected to perform a typical differential analysis (i.e., a very common approach in metagenomics). In both analyses, we submitted the raw FASTQ files and provided a target file containing sample information (needed for statistical analysis).

#### Zymo mock dataset

The mock sequencing (EBI ENA code PRJEB33737) of the ZymoBIOMICS^TM^Microbial Community DNA was performed with an Illumina MiSeq resulting in 12 samples of 257,000 ±85,000 (mean ± SD) sequences of 300-base-long paired-end reads. The composition of the Zymo mock community is known and is composed with 8 phylogenetically distant bacterial strains, 3 of which are gram-negative and 5 of which are gram-positive. DNA of two yeast strains that are normally present in this community were not amplified. Genomic DNA from each bacterial strain was mixed in equimolar proportions (https://www.zymoresearch.com/zymobiomics-community-standard). We compared the impact of both the number of amplification cycles (25 and 30 cycles) and the amount of DNA loaded in the flow cell (0.5ng and 1ng), on the observed microbial abundance. Each sample was sequenced 3 times (experimental plan provided in supplementary materials). Sequencing report provided by the sequencing facility indicated the presence of contaminants. To handle this issue, we filtered out the genera occurring in less than 12 samples and outliers with a reduced log abundance as compared to the other genera (Fig. S2).

#### Afribiota dataset

The second dataset included 541 samples of microbial communities in stunted children aged 2-5y living in sub-Saharan Africa (EBI ENA code PRJEB27868) [50]. Three groups (nutritional status) of individuals were considered: NN=non stunted, MCM=moderately stunted, MCS=severely stunted. samples originated from the small intestine fluids (gastric and duodenal) and feces. The authors performed the bioinformatic treatment with QIIME framework and the statistical analysis with several R packages including Phyloseq for the normalization and DESeq2 for the differential analysis. Using SHAMAN, raw reads were filtered against Human HG38 and PhiX174 genomes. A total of 2386 OTUs were calculated and 76% were annotated with SILVA database at genus level. The sparsity rate of the contingency table was high with 0.84. In consequence, we used the weighted non-null normalization which is particularly efficient when the matrix is highly sparse (Fig. S1). Two analyses were performed, a global analysis that included duodenal, gastric as well as feces samples and a more specific analysis including fecal samples only. Statistical models included the following variables, age, gender, country of origin and nutritional status.

#### Benchmarking

Last, we compared the running time performance of SHAMAN with five other web applications for metataxonomy studies (ASaiM, FROGS, MetaDEGalaxy and Qiita). For each web interface, we submitted the raw sequencing reads of the Zymo mock dataset and estimated the time to generate a BIOM containing the contingency matrix and OTU annotation. Qiita and MetaDEGalaxy were used remotely, FROGS was installed on galaxy.pasteur.fr and ASAIM was installed on Mac book pro with a core i7 6cpu with 32Gbytes of ram.

## Results and discussion

### User cases

Data analysis in SHAMAN may easily be done by users who are not familiar with command-line analyses. In this paper we analyzed two datasets.

**Fig. 2:**
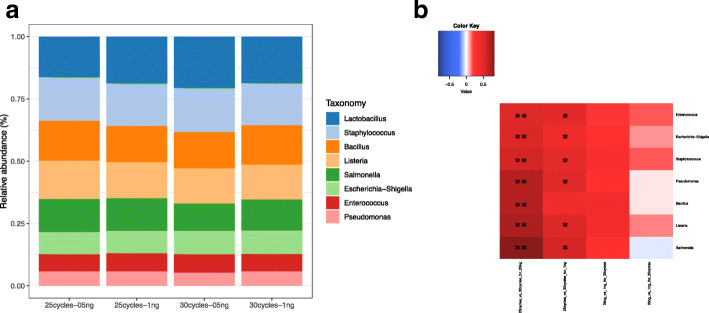
Taxonomic composition of the Zymo mock community as analyzed in SHAMAN. Samples were processed in the lab under two different type of treatment: varying amount of DNA (0.5 and 1 ng) and varying the number of PCR cycles (25 and 30 cycles). **a** Barplot of proportion of taxa in the different conditions. **b** Heatmap of log2 fold changes obtained in the different contrasts. ** indicates 0.001 <*p*-value <0.01; *, 0.01 <*p*-value <0.05

In the analysis of the Zymo mock dataset the 8 bacterial strains present in the samples were suitably detected (Fig. 2). We then defined a statistical model to test the effects of the two varying experimental factors: the amount of DNA and the number of amplification cycles and the interaction between th ese variables. The statistical comparison showed a stronger impact of the number of amplification cycles as compared to the amount of DNA. While we found no differential features between 0.5 ng and 1 ng DNA for each group of number of cycle (25 and 30 cycles), the comparison of the number of amplification cycles within each group of DNA amount revealed a significant impact on the observed abundance of mock bacteria (Tables S1, S2). These results are in agreement with previous studies that presented the PCR-induced bias on similar mixtures [51, 52].

**Fig. 3:**
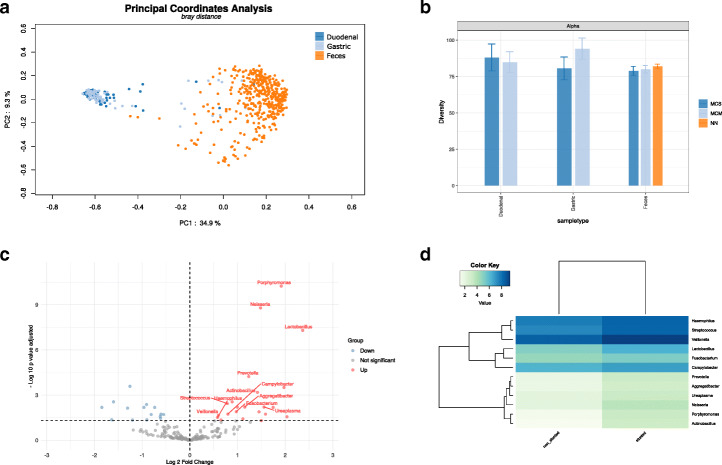
Afribiota study of small intestine fluids and feces from stunt children compared to non stunt. **a** PCoA plot the Bray-Curtis dissimilarity index of the samples. Duodenal samples are colored in blue, light blue for Gastric and orange for Feces. PERMANOVA test based on the sample type yielded a *p*-value of 0.001. **b** Alpha diversity analysis of non-stunt (NN), moderately stunted (MCM) and severely stunted (MCS). Overlapping confidence interval indicates that the diversity are not different between NN, MCM and MCS in duodenal, gastric and feces samples. **c** Volcano plot of differentially abundant genera in the feces of stunt children compared to non-stunt. We plot the log2 fold change against the -log 10 adjusted *p*-value. Microbial taxa in red correspond to an increase of abundance and in blue to a decrease abundance. Labeled dots correspond to taxa from oropharyngeal core microbiota. **d** Log 2 abundance of differential abundant taxa from oropharyngeal core microbiota in stunt and non-stunt children feces

Regarding the analysis of the second dataset with SHAMAN, overall our results were highly consistent with those of Vonaesch et al. [50]. We detected a significant change in the community composition between gastric and duodenal samples as compared to feces samples at Genus level (Fig. 3a) (PERMANOVA, P=0.001). The most abundant genera were reported in Fig. S3. *α*-Diversity was not affected by stunting (Fig. 3b). We looked for a distinct signature of stunting in the feces. In the volcano plot (Fig. 3c), we represent genera with differential abundance between stunt samples as compared to non-stunt (complete list available in Table S3). Twelve microbial taxa, corresponding to members of the oropharyngeal core microbiota, were over-represented in feces samples of stunted children as compared to non-stunted children. More particularly Porphyromonas, Neisseira and Lactobacillus (Fig. 3d) appeared more abundant. All those findings were in agreement with the conclusions of the Afribiota consortium while being obtained within a few minutes of interaction with the SHAMAN interface.

### Mapping SHAMAN among the other existing tools

To date, several tools have been developed for the analysis of metagenomic data. Relative to these existing tools, SHAMAN presents a number of interesting features and novelties. We made a brief qualitative assessment of the strengths and limits of SHAMAN, in comparison with other web interfaces designed for metataxonomic analyses (see Table 1). We first defined a list of important considerations that have practical implications for the user such as the possibility to process raw sequencing data, the existence of a statistical workflow and/or a visualization platform, possibility to store data and accessibility. For each web interface, we then evaluated whether it met those criteria. We believe that a nested solution, such as SHAMAN, is highly suited for producing robust and reliable results. Any results in SHAMAN may be cross-checked with a quantification or an annotation performed at an earlier stage.

**Table 1:** Mapping SHAMAN among the other web interface for metataxonomic analysis

**Category**	**SHAMAN**	**ASaiM**	**FROGS**	**MetaDEGalaxy**	**Qiita**	**Shiny-phyloseq**	**Metaviz**	**Vamps**
OTU processing	Yes	Yes	Yes	Yes	Yes	No	No	No
Computation time (min)	60	>1day	208	>1day	303	NR	NR	NR
Normalization	Yes	No	Yes	Yes	No	No	No	No
Modelling	Yes	No	*Manova*	Yes	No	D	M	No
Diversity analysis	Yes	No	Yes	Yes	Yes	*Alpha*	*Alpha*	*Alpha*
Phylogenetic analysis	Yes	No	Yes	Yes	Yes	Yes	No	*Tree*
Feature abundance plots	Yes	Yes	Yes	Yes	Yes	Yes	Yes	Yes
Ordination plots	Yes	No	Yes	No	Yes	Yes	Yes	Yes
Network plots	Yes	No	No	Yes	No	Yes	No	Yes
World-map distribution	No	No	No	No	No	No	No	Yes
Statistics plots	Yes	NR	No	No	NR	Yes	NR	NR
Interactive visualization	31	1	2;P	6	3	8	9	17
Raw data storage	No	No	No	No	Yes	No	No	Yes
Result storage	Yes	No	No	No	Yes	No	No	Yes
Online web Interface	Yes	No	No	Yes	Yes	No	Yes	Yes
R packaging	No	NR	NR	NR	NR	Yes	Yes	NR
Docker	Yes	Yes	No	No	No	No	Yes	No
Conda	Yes	No	Yes	No	Yes	No	Yes	No

D: Export from DESeq2, M: Export from Metagenomeseq, NR: Non relevant feature, P: Import/Export to Phyloseq, Computation time is indicated for the OTU processing of the Zymo mock communities, Number of unique interactive visualization are reported for each web interface in section ‘Interactive visualization’. We reported a specific implementation with the following terms: *Alpha* indicates when only alpha diversity is available for diversity analysis, *Manova* is indicated for FROGS because differential abundant features are detected with a Manova instead of a General Linearized Model. *Tree* indicates when no unifrac distance is available after computing the phylogeny of the OTUs

Comparison of computation time revealed that SHAMAN was faster than the other five web application to process the data of Zymo mock dataset. FROGS and QIITA are also convenient solutions for data processing since the whole OTU processing was performed in few hours. In both case, they provided an accurate description of the community as the 8 main communities composing the mock were correctly detected. On the other side, the computation time obtained with ASaiM and MetaDEGalaxy appeared much longer as we could not obtain a BIOM file after several days of calculation. Results obtained with Frogs and Qiita are reported on figshare (10.6084/m9.figshare.11815860). Furthermore several applications (as presented in Table 1), impose the burden of importing/exporting R objects which requires skills in R programming. This may also represent a source of reproducibility issues, notably in terms of compatibility of the packages over time.

## Conclusions

SHAMAN enables users to lead most of the classical metagenomics approaches. It also makes use of statistical analyses to provide support to each data visualization. The possibility to deploy SHAMAN locally constitutes an important feature when the data cannot be submitted on servers for privacy issues or because of insufficient internet access. SHAMAN also simplifies the access to open computational facilities, making a careful use of the dedicated server, galaxy.pasteur.fr.

Currently SHAMAN is limited to metataxonomic analyses. In a close future, we plan to extend our application to whole genome analysis, notably by using of microbial gene catalogs. Several catalogs are currently available to study human, mouse, cow, as well as ocean microbial diversity. A perspective will be to combine these results with metataxonomic data, and to perform integrative analyses.

During the development of SHAMAN, we felt a strong interest of the metagenomics community in our application. We recorded 82 active users per month in 2019 (535 unique visitors in total) and 800 downloads of the docker application. We expect that SHAMAN will help researcher perform a quantitative analysis of metagenomics data.

## Availability and requirements

Project name: SHAMAN Project home page: http://shaman.pasteur.fr, https://github.com/aghozlane/shamanOperating system: Platform independentProgramming language: ROther requirements: Python 3License: GNU GPL V3Any restrictions to use by non-academics: No

## Supplementary information

**Additional file 1** Supplementary materials (Appendix 1, Supplementary Figures S1-S3, Supplementary Tables S1-S2).

## Abbreviations

BIOM: Biological observation matrix
CV: Coefficient of variation
DNA: Deoxyribonucleic acid
EBI: European bioinformatic institute
ENA: European nucleotide archive
ITS: Internal transcribed spacer
MCM: Moderately stunted
MCS: Severely stunted
NN: Non stunted
NMDS: Non-metric multidimensional scaling
OTU: Operational taxonomic unit
PCA: Principal component analysis
PCoA: Principal coordinates analysis
PCR: Polymerase chain reaction
PERMANOVA: Permutational analysis of variance
RLE: Relative log expression
rRNA: Ribosomal ribonucleic acid
