# Supplementary material for: SHAMAN: a user-friendly website for metataxonomic analysis from raw reads to statistical analysis
Source: BMC Bioinformatics. 2020 Aug 10;21:345. doi: 10.1186/s12859-020-03666-4 (PMC7430814; doi:10.1186/s12859-020-03666-4)
Supplement: Supplementary file 1 — Additional file 1 Supplementary materials (Appendix 1, Supplementary Figures S1-S3, Supplementary Tables S1-S2). [file 12859_2020_3666_MOESM1_ESM.pdf]

Additional Files

Appendix 1: Mathematical definition of the contingency table filtering

Let us denote by $\mathscr{F}$ the entire set of OTU. We propose to only consider a subset $\mathscr{R}$ of OTUs, for the analysis: $\mathscr{R} = \mathscr{L}_1 \cap \mathscr{L}_2$, where $\mathscr{L}_1$ is defined by

$$\mathscr{L}_1 = \left\{ f \in \mathscr{F} \middle| \sum_j \mathbb{1}_{\{c_{fj}>0\}} \geq l_1 \right\} \quad \text{with} \quad l_1 = \lfloor 0.8 \times k_{max} \rfloor,$$

where c_{fj} is the abundance of the feature f in the sample j while k_{max} is the maximum number of samples in which the feature is found. The subset $\mathscr{L}_2$ corresponds to the features with a not too small abundance and is defined by

$$\mathscr{L}_2 = \left\{ f \in \mathscr{F} \middle| \sum_j c_{fj} \geq l_2 \right\},$$

where l_2 is the intercept of the linear regression between the variables $y_k = \sum_i \mathbb{1}_{\{\sum_j c_{ij} > x_k\}}$ and $x_k \in \left[\min_{f \in \mathscr{F}} \left(\sum_j c_{ij} \right) ; \lambda \right]$. λ is a tuning parameter whose default value is $\left\lfloor \frac{\sum_{ij} c_{ij}}{n} \times 0.05 \right\rfloor$.

Supplementary Figure 1: Boxplots of the average coefficient of variation for DESeq2, non-null, weighted non-null and Phyloseq normalization.

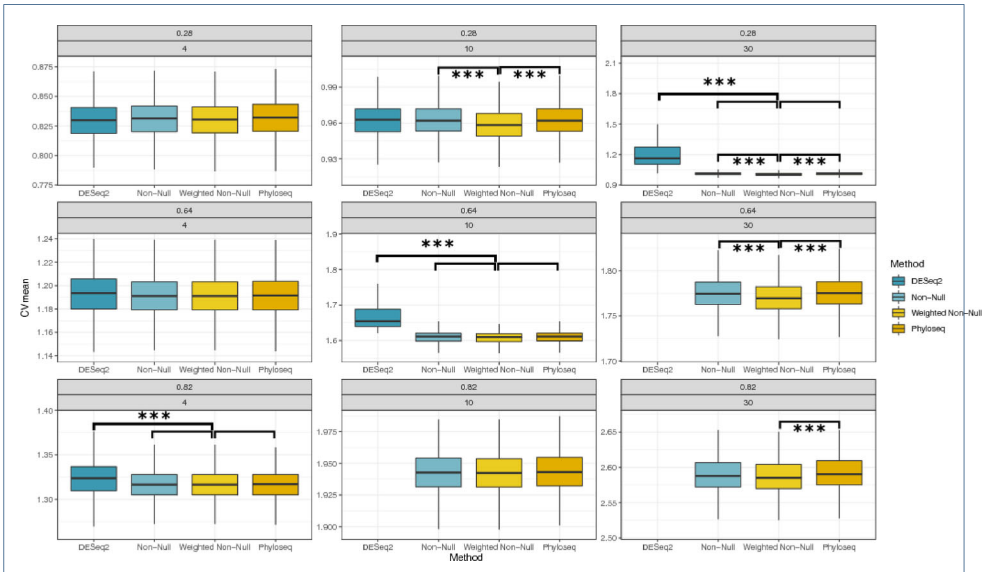

Figure S1 Boxplots of the average coefficient of variation for DESeq2, non-null, weighted non-null and Phyloseq normalization. The sparsity of the count matrices is chosen within $\{0.28, 0.64, 0.82\}$ and the number of observations vary within $\{4, 10, 30\}$. 500 normalizations were performed at each level of sparsity and for each number of observations. The results were analyzed by using a t-test, ***$p < 0.001$. DESeq2 normalization did not converged when the matrix sparsity and the number of observations was high (e.g., with a sparsity of 0.64 and 30 observations).

Supplementary Figure 2: Filtering of Zymo mock contaminant genera according to occurrence in samples and abundance.

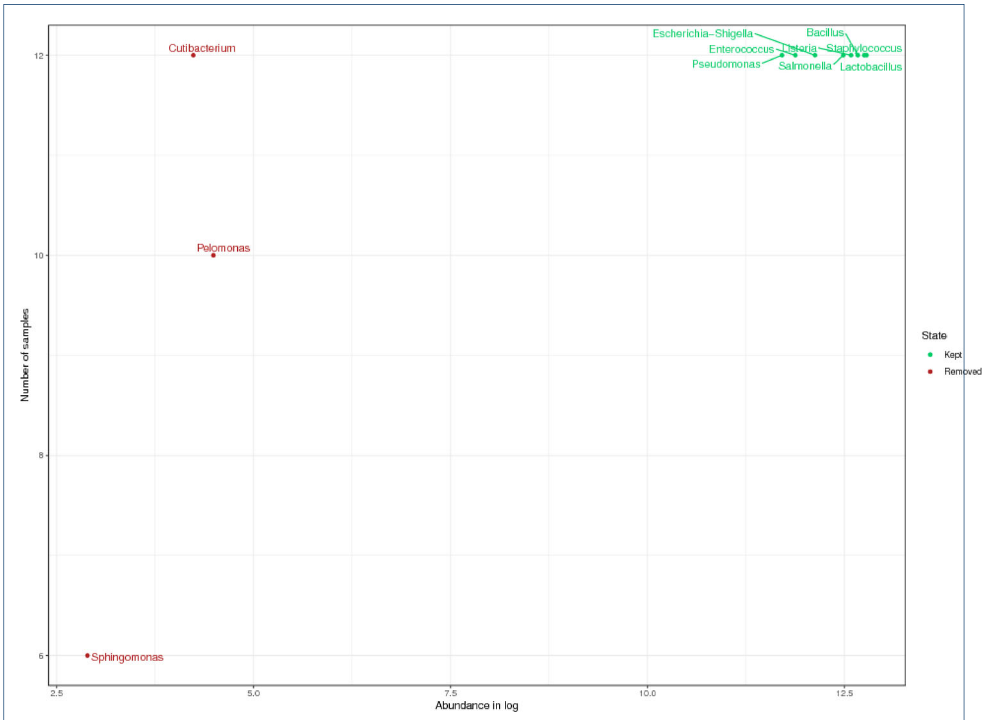

Figure S2 Filtering of Zymo mock contaminant genera according to occurrence in samples and abundance. Genera occurring in at least 12 samples and with log abundance $\geq$ 5 were kept in the study (green dots). Cutibacterium, Pelomonas and Sphingomonas genera counts (red dots) were removed from the contingency table.

Supplementary Figure 3: Barplot of the 12 most abundant genera in duodenal, gastric and feces from children of Central African Republic (CAR) and Madagascar.

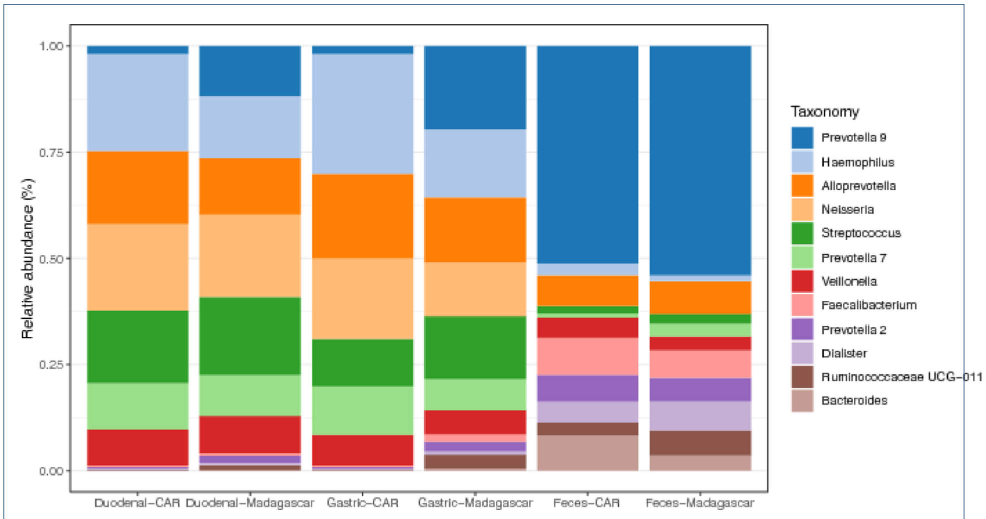

Figure S3 Barplot of the 12 most abundant genera in duodenal, gastric and feces from children of Central African Republic (CAR) and Madagascar. Haemophilus and Neisseria genera were also observed in cultured samples.

Supplementary Table 1: Summary of taxa differentially abundant when compared 25 and 30 amplification cycles for 0.5ng DNA load.

ID	Base mean	Fold Change	Log2 fold change	P-value adjusted
Bacillus	23314	1.613	0.69	0.0.0058
Listeria	22410	1.633	0.708	0.0.0058
Salmonella	19308	1.767	0.822	0.0.0058
Pseudomonas	8696	1.609	0.686	0.0045
Staphylococcus	26546	1.496	0.58	0.0045
Escherichia-Shigella	13921	1.432	0.518	0.0051
Enterococus	11024	1.417	0.504	0.0084

Table S1 Summary of taxa differentially abundant when compared 25 and 30 amplification cycles for 0.5ng DNA load Positive fold change indicates an increase of abundance of the taxa for the 25 amplification cycles.

Supplementary Table 2: Summary of taxa differentially abundant when compared 25 and 30 amplification cycles for 1ng DNA load.

Id	Base mean	Fold change	Log2 fold change	P-value adjusted
Enterococcus	11024	1.385	0.47	0.0303
Listeria	22410	1.427	0.514	0.0303
Salmonella	19308	1.473	0.559	0.0303
Staphylococcus	26546	1.381	0.465	0.0303
Escherichia-Shigella	13921	1.322	0.402	0.0393
Pseudomonas	8696	1.409	0.495	0.0414

Table S2 Summary of taxa differentially abundant when compared 25 and 30 amplification cycles for 1ng DNA load Positive fold change indicates an increase of abundance of the taxa for the 25 amplification cycles.

Supplementary Table 3: Summary of taxa differentially abundant when compared samples of stunted to non stunted children.

Id	Base mean	Fold change	Log2 fold change	P-value adjusted
Porphyromonas	4.73	3.772	1.915	5.755e-11
Neisseria	7.16	2.789	1.48	1.605e-09
Lactobacillus	51.69	5.152	2.365	4.962e-08
Prevotella	5.04	2.354	1.235	5.836e-05
Ruminococcaceae UCG-009	3.46	0.419	-1.255	0.0003
Weissella	11.33	3.927	1.974	0.0003
Actinobacillus	3.98	2.672	1.418	0.0006
Rikenellaceae RC9 gut group	86.21	0.33	-1.598	0.0028
Ruminococcaceae UCG-011	502.47	1.85	0.887	0.0028
Streptococcus	237.2	1.723	0.785	0.0036
Christensenellaceae R-7 group	49.22	0.534	-0.906	0.0042
Aggregatibacter	4.31	2.222	1.152	0.0061
Granulicatella	0.81	1.991	0.993	0.0061
Ureaplasma	5.52	2.94	1.556	0.0061
Ruminococcaceae UCG-002	144.01	0.601	-0.735	0.0065
Streptobacillus	0.63	3.351	1.745	0.0065
Terrisporobacter	4.29	0.514	-0.961	0.0065
[Eubacterium] xylanophilum group	2.05	0.405	-1.305	0.0073
Fusobacterium	28.55	1.964	0.974	0.0124
Abiotrophia	0.45	2.733	1.45	0.0126
Campylobacter	89.02	1.739	0.798	0.0181
[Eubacterium] coprostanoligenes group	119.89	0.657	-0.606	0.0181
Capnocytophaga	0.19	3.005	1.587	0.0181
Ruminococcaceae UCG-005	211.21	0.683	-0.55	0.0192
Ruminococcaceae UCG-010	31.58	0.57	-0.81	0.0192
Veillonella	475.74	1.515	0.599	0.0218
Ruminococcaceae NK4A214 group	36.02	0.651	-0.62	0.0257
Morganella	0.42	4.099	2.035	0.0271
Haemophilus	260.89	1.494	0.58	0.0301
Family XIII AD3011 group	9.67	0.653	-0.614	0.0344
Lactococcus	35.73	2.161	1.112	0.0373
Methanobrevibacter	5.99	0.321	-1.638	0.0429
Turicibacter	10.03	0.534	-0.9	0.0429
Escherichia-Shigella	25.03	1.574	0.654	0.0444
Kingella	0.47	2.822	1.497	0.0488

Table S3 Summary of taxa differentially abundant when compared samples of stunted to non stunted children. Positive and negative fold changes indicate respectively an increase of abundance of the taxa in stunted and non-stunted children.
